# Longitudinal changes in brain asymmetry track lifestyle and disease

**DOI:** 10.21203/rs.3.rs-4798448/v1

**Published:** 2024-08-08

**Authors:** Karin Saltoun, B.T. Thomas Yeo, Lynn Paul, Jorn Diedrichsen, Danilo Bzdok

**Affiliations:** 1 The Neuro - Montreal Neurological Institute (MNI), McConnell Brain Imaging Centre, Department of Biomedical Engineering, Faculty of Medicine, School of Computer Science, McGill University, Montreal, Canada; 2 Mila - Quebec Artificial Intelligence Institute, Montreal, QC, Canada; 3 Centre for Sleep and Cognition & Centre for Translational MR Research, Yong Loo Lin School of Medicine, National University of Singapore, Singapore; 4 Department of Medicine, Human Potential Translational Research Programme & Institute for Digital Medicine (WisDM), Yong Loo Lin School of Medicine, National University of Singapore, Singapore; 5 Department of Electrical and Computer Engineering, National University of Singapore, Singapore; 6 N.1 Institute for Health, National University of Singapore, Singapore; 7 Integrative Sciences and Engineering Programme (ISEP), National University of Singapore; 8 Martinos Center for Biomedical Imaging, Massachusetts General Hospital, Charlestown, MA, USA; 9 Division of the Humanities and Social Sciences, California Institute of Technology, Pasadena, CA, 91125, USA; 10 Western Institute of Neuroscience, Western University, London, Ontario, Canada

## Abstract

Human beings may have evolved the largest asymmetries of brain organization in the animal kingdom. Hemispheric left-vs-right specialization is especially pronounced in our species-unique capacities. Yet, brain asymmetry features appear to be strongly shaped by non-genetic influences. We hence charted the largest longitudinal brain-imaging adult resource, yielding evidence that brain asymmetry changes continuously in a manner suggestive of neural plasticity. In the UK Biobank population cohort, we demonstrate that asymmetry changes show robust associations across 959 distinct phenotypic variables spanning 11 categories. We also find that *changes* in brain asymmetry over years co-occur with *changes* among specific lifestyle markers. Finally, we reveal relevance of brain asymmetry changes to major disease categories across thousands of medical diagnoses. Our results challenge the tacit assumption that asymmetrical neural systems are highly conserved throughout adulthood.

## Introduction

It is hard to dispute that the brain has a role in controlling behaviour. Equally established is that both behaviour and environment led to fundamental structural adaptations in neural circuits (Zatorre et al., 2012). This interdependence between brain and lifestyle is perhaps not restricted to a critical period in early life. Rather, behavioral experiences influence brain structure throughout life (Boyke et al., 2008; May, 2011). Indeed, within-individual changes in brain architecture, as captured through structural brain scanning (T1-weighted MRI), can be observed after only 12 weeks of juggling practice, in young adults (Draganski et al., 2004), and middle-aged persons (mean age 60) (Boyke et al., 2008). Over the span of weeks, regular practice of a complex whole-body balancing task resulted in increased grey matter volume in left hemispheric regions including supplementary motor area, and medial orbitofrontal cortex; alongside simultaneous reductions in grey matter volumes in the right hemisphere, including right putamen, inferior orbitofrontal cortex and middle temporal gyrus (Taubert et al., 2010). In less than an hour of training, unskilled individuals learning to play piano exhibited task-specific structural modifications in diffusivity measures (DW-MRI) in left premotor area and left middle temporal gyrus (Tavor et al., 2020). Recent work has also advocated for the necessity/utility of dedicated longitudinal analyses to understand lifelong neuroplasticity, owing to the tendency of cross-sectional models to underestimate individual-level brain changes (Di Biase et al., 2023).

Behaviour-induced structural changes in adult brain structure are also evident in cognitive domains beyond motor capacities (Deary et al., 2010; Kanai & Rees, 2011; Zatorre et al., 2012). For example, both meta-cognition and language skills are responsive to regular active training. One month of mindfulness training resulted in within-individual white-matter changes in adults, including in the corpus callosum (Tang et al., 2010). Investigations conducted on identical twins showed strong evidence that white-matter structure inside the corpus callosum can be altered by specific environmental, rather than just genetic, factors (Chiang et al., 2009). Introduction of new lexical terms into the vocabulary of young adults was observed to induce diffusion-related structural modifications of cortical language areas, including left inferior frontal gyrus, left middle temporal gyrus, and left inferior parietal lobule (Hofstetter et al., 2017). These examples illustrate behavior-induced adaptations within the brain that involve both asymmetrical structural changes as well as alterations in the corpus callosum – the primary channel for information transfer between both brain hemispheres (Paul et al., 2007).

Habits and other regularly recurring behaviors in humans probably entail distinct manifestations in the brain. Empathy is an evolved inter-personal capacity which can broadly be distinguished into cognitive (‘I understand what you feel’) and emotional (‘I feel what you feel’) empathy (Shamay-Tsoory et al., 2009). Over the course of weeks, regular participation in training modules specifically designed to target and improve only one of these two different empathy systems resulted in longitudinal structural grey matter changes specific to the trained empathy system. Improvements in ‘cognitive empathy’ cooccurred with measurable changes in right middle temporal gyrus and left ventrolateral prefrontal cortex, but not their contralateral homologs. Conversely, training in ‘emotional empathy’ related to structural changes in regions including the right supramarginal gyrus, right insular-opercular regions, and left posterior cingulate cortex (Valk et al., 2017). Beyond week-long experiments, behavioral experiences potentially influence brain structure throughout the lifespan (Boyke et al., 2008; May, 2011).Language, in addition to being among the most sophisticated cognitive processes in humans, exhibits large degrees of asymmetrical brain specialization (Hartwigsen et al., 2021). In fact, in teenagers, growing proficiency in a second language occurring over the course of months was related to within-subject changes in grey matter density in the left inferior frontal gyrus and left anterior temporal lobe (Stein et al., 2012). Notably, absolute grey matter density was not linked to absolute language proficiency at any single timepoint; suggesting brain structure *changes* reflect language learning experiences, regardless of whether brain structure itself is associated with individual language skill (Stein et al., 2012). Thus, plastic changes in the brain can be specific to evolving mental capacities and may be sensitive to changes in a specific ability in a hemispherically differentiated manner.

Asymmetric lateralization of neurocognitive processes is a defining feature of many advanced mental abilities (Hartwigsen et al., 2021). Indeed, the association between cognitive impairment and localized regional structure measurements has previously been shown to be captured by the relative hemispheric balance between left-right homologs and not the total structural volume of either region of a pair per se (Cherbuin et al., 2010). In semantic dementia, the impacted hemisphere affects the type and degree of incurred cognitive deficits (Kumfor et al., 2016). As dementias progress, local asymmetries become more pronounced while remaining directionally stable (Haxby et al., 1990; Wachinger et al., 2016). However, across the human population, there is a co-existence between instances of both greater left hemisphere impact and greater right hemisphere impact (Kumfor et al., 2016). Therefore, aggregating across these instances of greater left hemisphere changes and greater right hemisphere changes may suggest that there is no preferred direction of change across individuals (Wachinger et al., 2016). Nevertheless, hemispherical asymmetries still exist on an individual level, and offer insight into progression of certain brain diseases.

Hippocampal asymmetry and its association with cognitive decline serves as a telling example for the interplay between structural asymmetry and behavioral measures. Looking into this region and its asymmetry, researchers have found that the magnitude of hippocampal volume asymmetry increases with the severity of neurodegenerative disease (Sarica et al., 2018). Another study of asymmetry of hippocampal morphological shape separately analyzed both amount of asymmetry (absolute hemispheric difference) and asymmetry (hemispheric difference). The authors found that dementia diagnoses were more strongly associated with absolute hemispheric differences than with the actual directional hemispheric differences (Wachinger et al., 2016). This was attributed by the authors to the lack of a dominant direction of hemispheric asymmetry across the population (Wachinger et al., 2016). In a separate analysis, the authors found longitudinal intraindividual factors captured two to five times more structural asymmetry change than cross-sectional age effects (Wachinger et al., 2016). This finding is consistent with the results of a more recent comparison of longitudinal versus cross-sectional brain trajectories, which found cross-sectional estimates of rate of change are insufficient to describe known longitudinal rates of brain change over time (Di Biase et al., 2023). Of importance to the present investigation, the authors argued that individual behavioural and cognitive measures may play a larger role than age in capturing observed brain feature changes (Di Biase et al., 2023).

Mechanistically, macroscopic changes observed through MRI can reflect a broad array of microstructural plasticity changes (Assaf, 2018). Invasive histological studies are largely limited to animal models and are ultimately necessary to link brain-imaging-derived measures of change to underlying cellular architecture (Caroni et al., 2012; Zatorre et al., 2012). Though few in number, existing histological studies in rats, mice, and monkeys pinpoint clear cellular substrates that underlie observed longitudinal changes in MRI signals (Zatorre et al., 2012). Mice trained on different kinds of the Morris water maze, each exercising different problem-solving and navigational skills, showed structural enlargement visible on MRI in the corpus callosum, striatum, and hippocampus. Each mouse training group exhibited distinct patterns of structural changes relevant to the cognitive approach to navigating their version of the water maze. Histological staining on these same mice, after training, showed that morphological brain changes coincide with GAP-43 protein expression: implicated in neural remodelling – a crucial component of the presynaptic terminal and axonal growth cones (Lerch et al., 2011). In another study on experimentally structured experience, rats which learned the location of a hidden platform in a water maze exhibited changes in fractional anisotropy (FA), a diffusion MRI-derived measure capturing white matter integrity, in the corpus callosum that was unseen in rats swimming in a water maze with no hidden platform. Histological staining revealed that changes in FA in the corpus callosum were associated with increased expression of myelin basic protein, a proxy of myelination (Blumenfeld-Katzir et al., 2011). In across-species investigations using comparable training programs for humans and rats, similar longitudinal structural changes across species were reported after completion of experimental tasks on short-term learning (Sagi et al., 2012). The magnitude of changes was associated with improvement in task performance, and histological staining in rats revealed changed expression of neuronal growth factors (BDNF) – encouraging growth and differentiation of new neurons and synapses – and synaptophysin, one of the most abundant synaptic vesicle membrane proteins, specifically in brain regions exhibiting structural changes (Sagi et al., 2012). Taken together, converging evidence suggests that macroscopic structural changes observed through MRI at different time scales can reflect cellular changes associated with synaptic vesicle formation and uptake, neuronal remodelling, and axon fiber myelination.

Previous studies on structural plasticity in both humans and model species have largely relied on targeted laboratory interventions to assess the relationship between pre-selected cognitive functions and brain structure. However, a strength of structural imaging is its ability to link individual’s lifestyle and behaviour in an ecologically valid environment, that is, everyday activities outside of the MRI scanner, to structural changes observed in MRI imaging (Kanai & Rees, 2011). Moreover, while plasticity has been reported to occur over the span of weeks (Boyke et al., 2008; Draganski et al., 2004), sometimes days (Taubert et al., 2010), and even hours (Hofstetter et al., 2017; Tavor et al., 2020), structural changes may persist over the course of years. Therefore, we propose an alternative approach towards naturalistic investigations into lifestyle events and habits and their correlates with within-individual changes. To this end, we leveraged the UKBB resource, which combines high-quality brain scans with concurrent real-world phenotyping measures of unprecedented depth. We built on previous measures of brain asymmetry which encapsulate consistent brain-global motifs of structural asymmetry, as derived from careful examination of the brains of over 37,000 individuals (Saltoun et al., 2023). We examined how the extent and expression of these brain asymmetry patterns change over the span of years in individuals. We capitalized on concurrent brain imaging and behavioural phenotyping conducted across years within the same individuals to specifically investigate how changes in global brain asymmetry, rather than global brain volume, relate to 977 demographic factors, behavioural measures, phenotypic characteristics across 11 domains. We further investigated how brain asymmetry changes relate to lifestyle and behavioural changes across 9 domains that co-occur alongside our discovered brain asymmetry changes. Additionally, by linking brain asymmetry to 4,448 total medical health record items, we related brain asymmetry changes to several major health outcomes. In a multi-prong approach, we considered both directional changes and absolute amounts of longitudinal brain asymmetry change across multiple brain asymmetry patterns, each of which individually captures distinct aspects of global brain asymmetry.

## Results

### The left and right brain hemispheres longitudinally change at different rates

**Figure 1:**
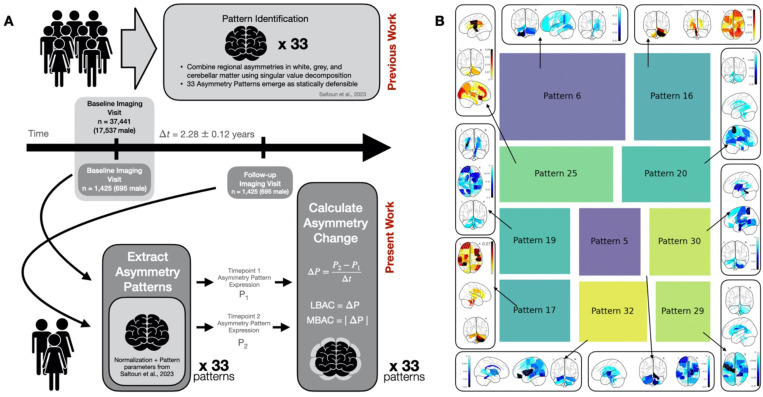
Systematic longitudinal changes occur in multiple structural whole-brain asymmetry patterns spanning white matter, cerebellum, and cortical structures (A) Two measure of structural brain asymmetry change both showcase relevant longitudinal progression. Amount of asymmetry change (MBAC) captures *how much* the structural imbalance between hemispheres progressed, regardless of direction. Asymmetry change (LBAC) captures *how* structural imbalance between hemispheres progresses, including which hemisphere showcases greater change. Mean LBAC and MBAC across all 33 statistically defensible asymmetry patterns. All asymmetry patterns concurrently consider brain features spanning cortical and subcortical grey matter, major white matter tracts, and cerebellar grey and white matter. All LBACs were compared in terms of relative right versus left grey matter cortical change to aid in interpretation. This reference composite reference measure tracks aggregate cortical asymmetry change but does not reflect the hemispheric bias of any individual grey matter homolog, or on the hemispheric biases of cerebellum or white matter tracts. (B) Asymmetry patterns exhibiting large asymmetry changes across the population draw upon distinct combinations of brain features spanning the whole brain. Brain feature contributions of the 10 asymmetry patterns with the largest LBACs. Square size represents relative amount of change amongst the top 10 pattern encapsulated by the given asymmetry pattern. Brain maps show cortical and subcortical; major white matter tracts, and cerebellum contributions to the specified asymmetry pattern. Brain features shown on the right hemisphere represent right hemisphere homologues exhibiting larger longitudinal shifts than left hemisphere counterparts in the positive direction, and the reversed configuration in the negative direction. Asymmetry patterns with negative mean directional change (LBAC) are illustrated with blue coloured brain maps. This corresponds to the reversed configuration of brain feature changes as delineated above, with brain features shown on the right hemisphere exhibiting diminished R>L asymmetry or equivalently increased L>R asymmetry with time.(C) and (D) The (Cohen’s d) effect of retirement status across multiple separate patterns is larger than the (Cohen’s d) effect of the pattern with the largest sex effect. Cohen’s d effect size of LBACs between sex or employment contrasts. Three employment Cohen’s d contrasts were conducted per asymmetry pattern, comparing participants who were in full-time employment at both time points (employed), in retirement at both time points (retired) or transitioned from full-time employment at first imaging visit and retired at second imaging visit (retiring). Grey rectangle indicates single largest absolute Cohen’s d for sex-contrast (pattern 21, |d|=0.176).

Here, we tested the possibility of longitudinal progression of brain asymmetry, alongside its associations with real-world lifestyle indices. The present investigation built on our recently established whole-brain asymmetry patterns, which capture concurrent left-right deviations from ~37,000 UKBB participants (Saltoun et al., 2023). The examination of the structural brain asymmetries unveiled 33 robust patterns of structural asymmetry which implicate distinct sets of brain features from across the brain, including white matter tracts, subcortical structures, and cerebellar lobules (Saltoun et al., 2023). We extended this investigation beyond a single snapshot in time into the current longitudinal examination of the question if and how structural asymmetry changes within individuals over years (Fig. 1A).

To reach this goal, we capitalized on all UKBB subjects with two brain-imaging visits, reaching a total of 1425 individuals (49% male; 27.4 ± 1.4 months between visits; 62.5 ± 7.2 years old at first imaging visit). As a preparatory analysis step, we probed for the possibility of unequal structural changes between pairs of homologous grey matter volumes. By assessing regional volumes over time, we found that longitudinal volumetric change in homologous grey matter regions only track each other incompletely over years. Across 55 homologous grey matter pairs, the volume change over time in the left homolog was not perfectly aligned with the volume change over time in the right counterpart (r = 0.51 (mean), ranging from r = 0.19 to 0.84 for all 55 grey matter regions). The lack of full correspondence between longitudinal volumetric changes in homologous pairs (i.e., rates of change were unequal between left and right hemisphere homologs) confirmed our intuition that longitudinal change in grey matter brain structure is a non-symmetric phenomenon in the human brain in general.

Motivated by the observation of hemisphere-specific rates of longitudinal change, as well as the recent insight that brain asymmetry is global rather than local phenomena (Saltoun et al., 2023), we elected to investigate structural brain asymmetry change through the lens of whole-brain structural asymmetry patterns (Fig. 1A). These asymmetry patterns holistically combine structural asymmetries from across the brain into composite measures which delineate specific motifs of structural asymmetry which respects the natural inter-dependence of brain parcels. First, we computed the expressions of all brain asymmetry patterns for all individuals with two time points using our publicly available asymmetry pattern definitions (Saltoun et al., 2023). Mathematically, asymmetry pattern definitions consolidate regional structural asymmetries, represented by lateralization indices $\left(LI=\frac{V_{R}-V_{L}}{0.5*\left(V_{R}+V_{L}\right)}\right)$, of 85 brain features (spanning 9 cerebellar parcels, 21 white matter tracts, 48 cortical grey matter regions, and 7 subcortical grey matter regions) into a single value which captures the expression level of a single asymmetry pattern in each participant and time point (Fig. 1B).

Once this “pertinence” of asymmetry patterns was calculated for both time points, a comparison across visits was conducted to examine if and how whole-brain asymmetry shifts in individuals across time. Taking into account previous studies indicating a lack of preferentially impacted hemisphere in some forms of brain change (Wachinger et al., 2016), we elected to describe structural asymmetry changes with two complementary measures. Namely, we concurrently investigated both lateralized brain asymmetry changes (LBACs) and absolute magnitude of brain asymmetry change (MBACs). LBACs were measured as $LBAC_{k}=\frac{P_{k_{2}}-P_{k_{1}}}{\text{Δ}t}$ where $P_{k_{t}}$ indicated asymmetry pattern expression at timepoints 1 $\left(P_{k_{1}}\right)$ and 2 $\left(P_{k_{2}}\right)$ while k indexed a particular asymmetry pattern k. LBACs captured consistent brain asymmetry changes across the population and are sensitive to instances where the hemisphere undergoing faster rate of longitudinal change for a particular homologous pair is shared across the population. For example, the planum temporale is heavily implicated in asymmetry pattern 2, and may undergo imbalanced structural change over time. If, across the population, the volume of the right planum temporale shrinks at a more rapid rate as compared to the left planum temporale, the observed asymmetry pattern 2 LBAC would be large indicating more extreme leftward planum temporale asymmetry at the follow-up timepoint. On the other hand, MBAC, measured as $MBAC_{k}=\frac{\left|P_{k_{2}}-P_{k_{1}}\right|}{\text{Δ}t}$, which captured the amount of asymmetry change independent of direction, is suited to capturing hemispherically-specific rates of change, even when distinct directions of asymmetry change co-occur across the population. Thus, if half of the population exhibits longitudinal increase of leftward asymmetry and the other half of the population exhibits similar increases in rightward asymmetry, the LBACs would be small (suggesting that the rate of change in the left and right hemispheres are similar). Yet, on the individual level, there is consistent skewing in one or the other direction. This would result in a measurable MBAC without a corresponding LBAC effect, highlighting the value of indexing change through both LBAC and MBAC in parallel.

By construction, our measures of asymmetry change (LBAC and MBAC) concurrently combine longitudinal changes across different levels of brain organization, including cerebellar tissue, white matter tracts, and cortical and subcortical volumes. To aid interpretation, we also related all LBACs to relative right versus left grey matter change. Based on evidence that onset of age-related volume change occurs earlier in grey matter than in white matter (Fotenos et al., 2005), we calculated a reference measure which captures which hemisphere exhibited larger cortical grey matter decrease. To do so, the yearly rate of change in cortical grey matter in the left $\left(\text{Δ}G_{L}\right)$ and right hemisphere $\left(\text{Δ}G_{R}\right)$ was computed, at which point the difference between observed volumetric change across hemispheres $\left(\text{Δ}G_{R}-\text{Δ}G_{L}\right)$ revealed which hemisphere exhibits faster volume changes. We found a common overall trend of volume declines, rather than volume gains, across the population for both hemispheres, with the right hemisphere exhibiting slightly slower volume declines $\text{Δ}G_{R}=-1243\pm 69\;{\text{mm}}^{3}/\text{year}$; standard error of mean (SEM)) than the left hemisphere $(\text{Δ}G_{L}=-1328\pm 64\;{\text{mm}}^{3}/\text{year; SEM)}$. To relate the overall cortical change to the LBACs, we computed the Pearson’s correlation coefficient between LBACs and hemisphere preferential longitudinal cortical volume change. Through this, we confirmed that positive LBACs correspond to the left hemisphere getting smaller faster than the right hemisphere.

### Distinct whole-brain asymmetry patterns show unique changes over several years

**Figure 2:**
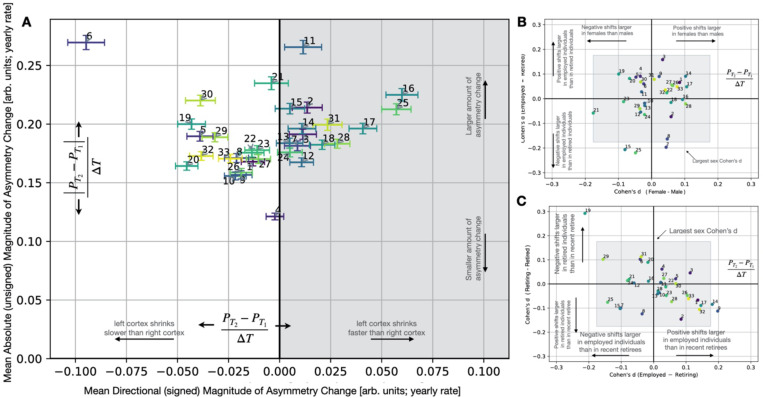
Major lifestyle events reflect in longitudinal progression of structural whole-brain asymmetry patterns (A) Two measure of structural brain asymmetry change both showcase relevant longitudinal progression. Amount of asymmetry change (MBAC) captures *how much* the structural imbalance between hemispheres progressed, regardless of direction. Asymmetry change (LBAC) captures *how* structural imbalance between hemispheres progresses, including which hemisphere showcases greater change. Mean LBAC and MBAC across all 33 statistically defensible asymmetry patterns. All asymmetry patterns concurrently consider brain features spanning cortical and subcortical grey matter, major white matter tracts, and cerebellar grey and white matter. All LBACs were compared in terms of relative right versus left grey matter cortical change to aid in interpretation. This reference composite reference measure tracks aggregate cortical asymmetry change but does not reflect the hemispheric bias of any individual grey matter homolog, or on the hemispheric biases of cerebellum or white matter tracts. (B) and (C) The (Cohen’s d) effect of retirement status across multiple separate patterns is larger than the (Cohen’s d) effect of the pattern with the largest sex effect. Cohen’s d effect size of LBACs between sex or employment contrasts. Three employment Cohen’s d contrasts were conducted per asymmetry pattern, comparing participants who were in full-time employment at both time points (employed), in retirement at both time points (retired) or transitioned from full-time employment at first imaging visit to retired at second imaging visit (retiring). Grey rectangle indicates single largest absolute Cohen’s d for sex-contrast (pattern 21, |d|=0.176).

We found that structural brain asymmetry is not static in adults from our UK Biobank cohort. Brain asymmetry (28 out of 33 examined patterns) exhibited robust LBACs over time (Fig. 2A). While 5 out of the 10 asymmetry patterns with the largest LBACs also were among the 10 patterns with largest MBACs, mean LBACs were relatively small compared to mean MBACs. This suggests that although individual brains change a lot in their asymmetry, across the population there may be a less consistent direction which becomes relatively larger or smaller. Indeed, all patterns showed salient MBACs, even asymmetry patterns with no mean change in LBAC across the population. For example, asymmetry pattern 21, which combines ipsilateral middle frontal gyrus and amygdala shifts with contralateral shifts in paracingulate gyrus and corticospinal tract, exhibited a mean LBAC consistent with zero across the population. This isolated observation may be taken to suggest that this particular asymmetry pattern exhibits modest longitudinal changes towards a particular hemisphere. However, this same asymmetry pattern (21) displayed the third largest MBAC amongst all our studied asymmetry patterns. Therefore, though *individually* this measure of asymmetry changes significantly with time, across the population there are individuals whose asymmetry pattern moves in the opposite direction.

As a reminder, our LBACs did not capture the total hemispheric volume decline or gain. Instead, the grey/white matter imbalances between hemispheric homologs across the whole brain and the way in which these left-right deviations evolve with time within individuals. As indicated by its small MBAC (i.e., total asymmetry change), asymmetry was most stable for pattern 4 (Fig. 2A), which highlighted several language-related brain regions. Pattern 4 was characterized by asymmetrical bias to the same hemisphere in inferior frontal gyrus, pars triangularis and frontal operculum cortex alongside asymmetries which favour the contralateral hemisphere in planum polare and central opercular cortex. Furthermore, LBAC for pattern 4 overlaps with zero, indicating there is no preferred left-ward or right-ward direction of asymmetry change for this pattern at the population level.

In contrast, overall, pattern 6 showed the most prominent plastic asymmetry with time (largest MBAC, Fig. 2A). This pattern strongly emphasizes cerebellar asymmetries, with an emphasis on cerebellar lobule VIIIa, 8b, 9 asymmetries in the same direction, alongside ipsilateral shifts in language-related cortical grey matter features including the supramarginal gyrus and contralateral central operculum cortex (Fig. 1B). In addition, pattern 6 also exhibited the largest directed left-right shift (LBAC) across all examined asymmetry patterns, suggesting a common motif across the population wherein cerebellar homologs become more left biased with time. The directional trend of asymmetry pattern 6 progression (LBAC) was associated with greater right hemispheric (versus left) cortical volume decline.

Taken together, brain asymmetry patterns with consistently large magnitude (MBACs) did not necessarily show consistent direction (LBACs) of change over time. However, patterns which exhibited large MBACs consistently draw upon distinct and complementary brain locations of left-right divergence. By contrast, the two patterns with largest LBACs both drew upon cerebellar regional masses: pattern 6 (largest LBAC) emphasizes lobes 8, 9 and crus I and II; asymmetry pattern 16 emphasizes cerebellar lobes 6, 7b and crus 2. Pattern 16 emphasized concurrent cerebellar shifts with white matter asymmetries, including asymmetries in cerebellar and cerebral peduncle, medial lemniscus, and corticospinal tract. Instead, in pattern 6, cerebellar asymmetries systematically related to cortical regions rather than white matter tracts.

### Large asymmetry changes occur in both directions throughout adulthood

Each asymmetry pattern captured a distributed set of brain features at the population level which exhibited linked deviations from symmetry across levels of brain organization. We also confirmed that LBACs and MBACs describe separate reasons behind asymmetry changes, as noted by the weak correlation between LBACs and MBACs (mean |r| of 0.04 ± 0.03) across our UKBB participants (Supp. Fig. 1C). LBAC and MBAC of the same pattern showed the relatively strongest correlation (mean |r| of 0.13 ± 0.08). The complementarity of our separate measures of brain asymmetry change suggests that large absolute asymmetry changes (MBACs) can occur regardless of direction of change (as captured by LBACs). We also note that MBACs are weakly coupled with one another (|r| of 0.12 +− 0.06 (mean + std), with maximum |r| of 0.45 between MBACS in pattern 2 & 3), suggesting that brain asymmetry exhibits appreciable changes in adulthood across the whole brain in multiple distinct ways (Supp. Fig. 1C). These observations add bricks of evidence to structural brain asymmetry as a dynamically shifting property of human brain organization.

By construction, asymmetry patterns integrate structural brain asymmetries across the brain (based on singular value decomposition), and are linearly independent from one another at the first measurement time point (Saltoun 2023). Yet, we revealed that the longitudinal trajectories of these distinct patterns nevertheless exhibited interdependencies. The evidence of these interrelationships between distinct asymmetry patterns, over time, suggests that asymmetry patterns are potentially subject to similar driving factors which result in observed trend of changes in one asymmetry pattern to be linked to changes in other patterns. However, the interrelationship between different patterns is not driven by a joint emphasis on the same constituent brain features.

For example, directional (lateralized) progression over time of pattern 2 and pattern 3 expression (LBACs) exhibited the strongest observed relationship across all examined interrelationships (anti-correlation of r = −0.66). Both patterns 2 and 3 implicate planum temporale asymmetry change as a driving contributor to the overall asymmetry pattern. However, both asymmetry patterns call upon the planum temporale such that greater left versus right cortical decline would result in a positive-valued LBACs in both patterns. Yet, positive-valued pattern 3 LBACs were linked to negative valued pattern 2 LBACs in the same individual. Thus, the relationship between pattern 2 and 3 LBACs is perhaps driven by the concurrent asymmetry shifts in distinct brain features, such as cerebellar lobules VI and VIIb in pattern 2 and the hippocampus and Heschl’s gyrus in pattern 3.

Linked brain asymmetry changes also occurred in the absence of overlapping brain features. For example, the second largest absolute correlation between asymmetry pattern changes is the relationship between changes in patterns 3 and 8 (LBACs, r = −0.51). Pattern 3 drew upon ipsilateral asymmetry changes in the planum temporale, superior temporal gyrus, Heschl’s gyrus, and hippocampus. Pattern 8 drew upon ipsilateral asymmetry changes in inferior and middle temporale gyrus, temporal fusiform cortex, pars triangularis of inferior frontal gyrus alongside contralateral changes in superior temporal gyrus, temporal pole and occipital fusiform gyrus. Of the top 10 brain atlas features describing pattern 3, none were among the top 10 features describing pattern 8. In fact, the planum temporale, which was the single strongest driving feature of pattern 3, was the second smallest contributor to pattern 8 expression (84^th^ out of 85 constituent brain features).

In summary, asymmetry patterns are linearly independent from one another at the first measurement time point, yet we observed interdependencies between longitudinal changes of these distinct patterns. This observation suggests potential common underlying motifs governing how brain asymmetry changes within an individual over time. Additionally, LBACs and MBACs are largely unrelated from each other, indicating that a double pronged approach to investigating brain asymmetry change opens a more holistic window onto how hemispheric imbalance progresses within and across individuals.

### Brain asymmetry changes explain total brain volume change better than age and sex

Next, we examined the relationship between total brain volume change – a commonly studied, global measure of brain structure progression – and our measures of asymmetry pattern change. Middle and late adulthood (age 35+) is typically associated with overall brain volume loss, with healthy individuals over age 60 showing steady brain volume losses of >0.5% per year (Hedman et al., 2012). We use L2-penalized linear regression models to predict overall brain matter change across different tissue types given exclusively longitudinal asymmetry pattern information (either LBAC or MBAC). These results were compared by competition against baseline models that only have access to information about age and sex (including common covariates: age^2^, age*sex, age^2^*sex). Age and sex are usually among the strongest obtainable effects in human brain biology in general and in structural brain scans in particular (Cole & Franke, 2017; Driscoll et al., 2009; Franke et al., 2010; Kiesow et al., 2020; Ritchie et al., 2018; Taki et al., 2011) and may be meaningfully tied to changes in brain volume (Blinkouskaya & Weickenmeier, 2021; Driscoll et al., 2009).

Importantly, asymmetry-pattern models (LBACs, MBACs) outperformed analogous age-sex models in explaining total brain volume progression between participants (Supp Fig. 2). In contrast, when considering overall brain volume at baseline, rather than its change, asymmetry-pattern models (LBACs, MBACs) performed worse than analogous age-sex models (Supp Fig. 3). We also observed better performance in explaining total brain volume change when considering asymmetry pattern progression instead of age and sex persisted when taking into account different types of brain tissues – grey matter only, white matter only, or combined grey and white matter. The ability of lateralized brain asymmetry changes to explain gross brain volume change is most apparent when assessing change in total white matter volume. Hence, models with access to information about either LBACs or MBACs were consistently able to track changes in total brain volume across all types of brain mass assessed (Supp Fig. 2).

We reason that, if changes in brain structure – either overall growth or overall decline — occur at an equal rate in both hemispheres, individual divergences in the relative rate of decline or growth between hemispheres, as captured by LBACs and MBACs, would not provide additional information on overall structural brain change. On the other hand, if brain changes occurred at a different pace in each homolog, measures capturing the relative difference in rate of change across hemispheres, such as LBACs and MBACs, would be able to capture total structural brain change – as encountered in our analyses. Overall, changes in total brain volume are a reflection of added-up effects from longitudinal changes in structural brain asymmetry of spatially distributed brain features. These findings attest to the biological meaningfulness and strength of effect of our examined longitudinal asymmetry pattern measures.

### Sex and age tie into how much, but not how, brain asymmetry is progressing

Both sex-divergent and age-sensitive cognitive processes are known to be tied to structural asymmetries. Biological sex plays a salient role in cognitive processes spanning from language, to viso-spatial reasoning, to social reasoning (Hirnstein et al., 2019; Kiesow et al., 2020; Nisbett et al., 2012). We here explored the role of sex and age in longitudinal changes in brain asymmetry, starting with *how* asymmetry patterns reconfigure over time (LBACs). Among our collection of examined asymmetry patterns, the longitudinal progression in brain asymmetry was most starkly different between males and females in asymmetry pattern 21 (Cohen’s d = −0.176, cf. above), followed by pattern 17 (Cohen’s d = 0.106) which tracks ipsilateral cerebellar lobule VIIIb, parietal operculum cortex and inferior temporal gyrus asymmetries (Fig. 1B).

Age at baseline played a large effects in directional longitudinal progression of brain asymmetry across multiple patterns (Supp Fig. 1A), which is different from cross-sectional aging effects, including pattern 20 (Cohen’s d = 0.219), pattern 6 (Cohen’s d = 0.156), and pattern 16 (Cohen’s d = −0.130). Pattern 21 was the most dissimilar between the sexes (Cohen’s d = −0.176) yet longitudinal progression of this asymmetry motif appeared similar in relatively younger and relatively older individuals (Cohen’s d = 0.005). To recap, asymmetry pattern 21 combines ipsilateral asymmetries in grey matter regions including middle frontal gyrus, amygdala, and paracingulate gyrus with asymmetries in contralateral white matter tracts including posterior corona radiata, medial lemniscus, and corticospinal tract. The Cohen’s d sex contrast indicated that male participants on average exhibited stronger asymmetry shifts corresponding to a left cortex undergoing larger longitudinal volume declines than the right cortex, compared to females. The result of the Cohen’s d analysis indicates a relative separation between the male and female UKBB participants but does not directly convey the mean asymmetry change in either group. When examining the mean LBAC change in males and females respectively, we found that the mean pattern 21 in males is positive (indicating a left cortex shrinking faster than the right cortex), whereas the mean LBAC in females is negative (indicating the right cortex shrinking faster than the left cortex) (Supp Fig. 1A). That is, the mean female exhibited pattern 21 asymmetry shifts in the opposite direction as the mean male. The number of males (n =695) and females (n = 730) is relatively balanced in the cohort. The combined effect of similar magnitude of asymmetry changes (MBACs) across sexes but in opposite directions may contribute to the observation of an across-population mean LBAC consistent with zero in pattern 21 (Fig. 1A). Nevertheless, this motif of sex-dependent longitudinal progression of asymmetry seems limited to the sex-dependent asymmetry pattern 21. Only three other asymmetry patterns (patterns 4, 14, and 15) exhibited LBACs in opposite directions in males and females, with absolute sex-contrast Cohen’s d ranging from 0.034 (pattern 4) to 0.102 (pattern 14). Overall, we found asymmetry pattern progressions in general occur in the same direction (LBAC in same direction) between sexes across most examined patterns.

Sex-specific longitudinal trajectories in asymmetry pattern changes could not be entirely attributed to sex differences in initial asymmetry pattern expression at baseline. Among our catalogue of 33 examined asymmetry patterns, 25 patterns exhibited significant sex-related divergences in expression at time point 1, without considering longitudinal brain change (Saltoun et al., 2023). Here now, the Cohen’s d analysis of the sex-related divergences in asymmetry pattern *changes* (LBACs) for each of these 25 patterns yielded absolute Cohen’s d ranging from 0.021 (pattern 13) to 0.176 (pattern 21). Asymmetry pattern 6 exhibited the largest sex-divergence in pattern expression at baseline (Saltoun et al., 2023), yet revealed small sex differences in the longitudinal progression of pattern expression (Cohen’s d = −0.024; Fig. 2B). Overall, we found modest differences in the changes in hemispheric balance in males and in females even in asymmetry patterns where baseline asymmetry was significantly different between the sexes.

Subsequently, contrasting asymmetry magnitude changes (MBACs), our results revealed a consistent trend for males to show larger absolute asymmetry changes than females. MBACs in males exceeded those in females in 29 out of 33 asymmetry patterns. We also founnd that particular motifs of hemispheric asymmetry appear particularly sensitive to biological sex, providing hints that demographic characteristics may coincide with specific asymmetry changes. Asymmetry pattern 6 showed the largest asymmetry magnitude difference in males compared to females (Cohen’s d = −0.240), indicating that the rate of asymmetry changes in males exceeds that in females. Of the 4 asymmetry patterns where change magnitude in females exceeded that of males (patterns 20, 23, 27, 32), Cohen’s d effect sizes were modest, ranging from 0.032 to 0.050. By contrast, the trend for males to exhibit larger gross amounts of asymmetry change compared to females extended across a greater number of asymmetry patterns, and exhibited larger effect sizes (Supp Fig. 1D). Taken together, the larger effect size of sex divergences in MBACs as compared to LBACs indicates sex impacts how *much* brain asymmetry changes, more strongly than it impacts *how* brain asymmetry changes. This could be a potential reason for why larger brain asymmetries are observed in males rather than females.

Next, to investigate the role of age in the longitudinal progression of whole-brain asymmetries, the cohort was partitioned by age brackets, and the longitudinal brain asymmetry progressions were contrasted between the 25% oldest participants at baseline (age >= 69 years old; n = 342) and the 25% youngest participants at baseline (age <= 58 years old; n = 345). The effect sizes separating the LBACs of the relatively older and relatively younger participants were modest (mean absolute effect size d = 0.0649 +− 0.0504 across 33 patterns). Relatively older individuals displayed larger shifts in the asymmetries of brain features including cerebellar lobules 8 and 9 alongside lingual gyrus and posterior supramarginal gyrus compared to relatively younger individuals. Examining the absolute amount of change in relatively older compared to younger adults revealed a consistent motif of faster rates of brain asymmetry change in relatively older individuals (Cohen’s d < 0 in 32 examined patterns, associated with larger MBACs in older adults). Age-related effect sizes in MBACs (mean absolute effect size d = 0.176 +− 0.079 across 33 pattern MBACs) consistently exceeded effect sizes in LBACs. Age was associated with larger effect sizes in comparison to sex.

Overall, we found that both age and sex are meaningfully tied to longitudinal progression of global brain asymmetry (both LBACs and MBACs); with age being the more relevant factor for both measures of global brain asymmetry progression. Put differently, both age and sex were tied to how *much* brain asymmetry changes (MBAC) more strongly than they are tied to *how* brain asymmetry changes (LBAC).

### Transitioning into a new phase of life shows brain asymmetry reconfigurations

Investigations into structural brain changes to date have repeatedly highlighted the role of regular training and skills development as precursors to plastic brain adaptations, as measurable by MRI technology, even over the course of weeks (cf. Introduction). Here, 2–3 years elapsed between consecutive structural brain scans, over which time larger changes in lifestyle and behaviour may potentially occur. Because structural brain scan measurements, unlike functional brain scans, are not influenced by in-scanner tasks or activities, it has been argued that the resultant neuroanatomical measures are particularly well suited to capturing brain-correlates of lifestyle factors measured in ecologically valid (non-scanner) scenarios (Kanai & Rees, 2011). In this spirit, we turned to the onset of retirement (a lifestyle factor) to carry out a naturalistic study, or quasi-experiment, on how a major transition from one life phase to another may ignite plastic structural brain changes.

Leaving the workforce for retirement marks an important inflection point in individuals’ lives. This step has wide-ranging repercussions on social environment, sense of purpose, and daily environmental exposures. Therefore, we confronted retirement as a prime target to organically investigate how major life events may coincide with reconfigurations of structural brain asymmetry. We considered i) individuals in full-time employment at both imaging visits (n = 870 UKBB biobank participants), ii) individuals in retirement at both imaging visits (n = 308), and iii) individuals who were transitioning between these two life phases in the years between imaging visits (n = 121; henceforth *retiring*). To analyze how such distinct demographic statuses relate to brain asymmetry change, we computed Cohen’s d group differences in three separate contrasts of employment status pairs. We found effect size strengths across all employment contrasts consistently exceeded the largest effect size of the most sex-dependent patterns (cf. above. This observation suggests that tangible lifestyle factors may have more prominent relationships with brain asymmetry reconfigurations than more commonly studied dimensions such as sex. Our collective findings (LBAC effects > MBAC effects) suggested that within-subject brain changes dependent on employment status may be especially tied to *direction,* rather than *magnitude,* of hemispheric asymmetry shifts.

Retirement emerged as a salient feature describing re-arrangements in several of our asymmetry patterns. Six separate asymmetry patterns exhibited absolute Cohen’s d >0.175, the level of the strongest sex differences in our study (cf. above), in at least one contrast (Fig 2B and C). In 3 of the 6 most retirement-sensitive patterns, as expected, the most relevant employment contrast occurred when comparing brain morphology changes in individuals in full-time employment at both time points to those in full-time retirement at both time points. These asymmetry patterns drew upon largely distinct local shifts. Full-time employment versus retirement distinguished longitudinal shifts in asymmetry pattern 25 (employed – retired Cohen’s d = −0.220; Fig. 2B), which drew upon asymmetries in white matter fibers including ipsilateral shifts in cerebral peduncle, posterior internal capsule and superior corona radiata; and also implicated asymmetries in ipsilateral pallidum and contralateral ventral striatum (Fig. 1B). Additionally, the retirement versus employment contrast flagged pattern 15 (employed – retired Cohen’s d = −0.206; Fig. 2B) and pattern 7 (employed – retired Cohen’s d = −0.197; Fig. 2B). Pattern 15 drew upon structural asymmetry changes particularly in cortical regions, including ipsilateral angular gyrus, subcallosal cortex and contralateral postcentral gyrus; as well as crus 1 of the cerebellum and ventral striatum. Overall, distinct modes of life (full-time employment versus long-term retirement) reverberate in longitudinal structural brain changes across spatially distributed brain features in an asymmetric manner.

We next shifted focus from the contrast between static work environments (employed against retirement) to the transition into retirement itself in our UK Biobank participants. We observed the largest retirement-related effect size across all contrasts (33 patterns x 3 retirement contrasts) in pattern 19’s change directions (LBACs) when contrasting retiring individuals (whose retirement occurred in the 2–3 year interval between consecutive imaging visits) versus retired individuals (whose retirement occurred prior to the first imaging visit). Pattern 19 draws upon asymmetries in ipsilateral temporal occipital fusiform gyrus, middle frontal gyrus and anterior parahippocampal gyrus, alongside concurrent contralateral shifts in tapetum, and posterior thalamic radiation fiber tracts (Fig. 1B). The longitudinal trajectory of pattern 19 expression appeared more similar in retired versus employed individuals (employed – retired Cohen’s d = 0.100). Taken together, our contrast analyses suggested that the onset of retirement coincided with longitudinal reconfigurations of brain asymmetry in a distinct way. Beyond pattern 19, we found that the transition to retirement (contrasting full-time employment) yielded effects in asymmetry patterns 9 (Cohen’s d = 0.198; Fig. 2C), which highlights asymmetries in cerebellum, fusiform cortex, and supramarginal gyrus, as well as pattern 14 (Cohen’s d = 0.181; Fig. 2C) with asymmetry effects in frontal pole, and medial lemniscus. The collective findings suggest that the very act of retirement - a major life transformation – tended to co-occur with dedicated left-right hemisphere shifts in the whole brain.

Subsequently, we compared magnitude against direction properties in these progressions of hemispheric re-organizations. We consistently observed that direction effects (LBACs) were larger than magnitude effects in brain asymmetry change (MBACs). In contrast to sex and age effects (cf. above), retirement resonates more with *how* asymmetry changes rather than *how much* asymmetry patterns change. Nevertheless, our MBAC analyses shed light on how the various employment states influence brain asymmetry change. Consistently across patterns, the absolute rate of brain asymmetry change (MBAC) is faster in individuals who are retired as compared to individuals who are employed (employed – retired Cohen’s d contrast is < 0 in 28 out of 33 patterns; Supp Fig. 1D). Individuals who are retired also displayed faster absolute rates of asymmetry change than individuals who are retiring (retiring – retired Cohen’s d < 0 across 25 patterns; Supp Fig. 1E; for more details also see Supp Fig. 4).

Taken together, the act of retirement marks an incisive turning point in life, which may also help neuroscientists see deeper into tectonic progression of whole-brain structural asymmetry. We found retirement – and its contemporaneous revision in living environment – on the whole to be especially closely tied into *how*, rather than how much, asymmetry patterns progressed. Different life stages impacted distributed sets of local asymmetries in distinct ways. Notably, retirement-related asymmetry effect sizes exceeded those of sex effects, suggesting that lifestyle may explain more variation in within-subject asymmetry progression than more commonly studied demographic factors.

### Day-to-day lifestyle and cognitive performance are reflected in brain asymmetry changes

Next, we put the real-world relevance of the delineated asymmetry pattern trajectories to the test. Employment status change is only one of a variety of candidate changes in behavior and environment that potentially evoke neural processes that contribute to plastic remodelling. To gain a phenome-wide synopsis of how *changes* in various lifestyle indicators may resonate in within-individual *changes* of whole-brain asymmetry motifs, we conducted L2 penalized linear regression models predicting asymmetry pattern changes based on a rich array of behavioural/lifestyle phenotypes and phenotype changes (cf. Methods). Our 959 phenotypes spanned a total of 11 domains as measured at initial UKBB visit, ranging from mental health to cardiac measures, to blood assay results to early life factors. For brevity, we refer to this collection of phenotypes as lifestyle and behavioural factors. Phenotype *changes* captured difference in questionnaire responses or physical assessment as assessed at each imaging visit. Within each domain, a model estimating brain asymmetry change was constructed integrating one domain of either baseline or change in phenotypes alongside to demographic indicators (age, sex, age-sex covariates) and time between visits. To confirm the added value of considering lifestyle variables from these demographic indicators in assessing brain asymmetry change, we compared against base models containing only age, sex, age-sex covariates, and time between visits with no additional lifestyle phenotypes (cf. Methods). In so doing, we have systematically charted how a palette of everyday life indicators reverberate in longitudinal brain asymmetry changes.

**Figure 3:**
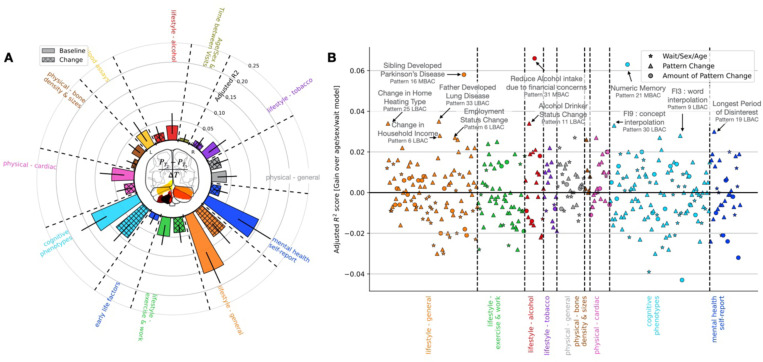
Changes in lifestyle and cognition reflect changes in structural brain asymmetry A (A) Specific behavioural domains capture longitudinal progression of brain asymmetry. Coefficient of determination (R^2^) of L2 penalized linear regression models predicting pattern 6 LBACs on the basis of behavioural phenotypes within a given domain. All models additionally contained sex, age at first imaging visit, and time between visits information. R^2^ scores were adjusted for number of parameters in the models. Phenotype changes represent the difference in response variables between the first and second imaging visits, and do not contain information about baseline response. Cerebellar contributions to pattern 6 are showcased in the centre of the graph. Ipsilateral cerebellar lobule VIIIa, 8b, 9 shifts alongside contralateral cerebellum crus I and II shifts characterize asymmetry pattern 6. (B) Whole-brain asymmetry pattern changes reflect changes in lifestyle and behaviour. Coefficient of determination (R^2^) gain derived from including LBAC and MBAC information in L2 penalized linear regression models predicting a behavioural phenotype on the basis of non-brain variables as well as brain asymmetry changes. Baseline comparison models contained only information about age, sex, and time between visits. R^2^ scores were adjusted for number of parameters in the models. A separate regression model was constructed for each phenotype. Shape of points represent the largest absolute coefficient contributing to the behavioural prediction, categorized according to membership to LBAC (triangle), MBAC (circle) or non-brain variables (age, sex, time between visits; star). Measures of socioeconomic status, including change in household income and employment status feature among phenotypic changes linked to structural brain asymmetry change.

As a recurring tendency, changes in asymmetry patterns were explained by mental health self-report markers, cognitive performance, and lifestyle indicators (Fig. 3A). This trifecta persisted across measured kinds of brain asymmetry change (MBACs and LBACs) and across examined patterns (Supp Fig. 5). Additionally, specific asymmetry patterns were characterized by distinct sets of lifestyle and behavioural indicators. Adding to its relationship with retirement (cf. above), pattern 19’s change directions (LBACs) were related to both baseline and changes in measures of socioeconomic status, including educational attainment, local employment and crime scores. In contrast, asymmetry pattern 16 was linked to baseline measures of socioeconomic status only, namely household income and local greenspace and pollution measures. Asymmetry pattern 16 was linked to changes in life stressors such as the development of illnesses or death in close family members. Hence, distinct lifestyle factors related to separate brain asymmetry progression.

To further summarize consistent motifs of lifestyle which linked to brain asymmetry progression in a principled approach, we gained insight estimating a domain-level model of the obtained brain asymmetry change-specific model fits (cf. above in this section). We applied principal component analysis on the coefficients of derived behaviour-brain models (L2-penalized linear regression models). Across asymmetry patterns, living in a rural versus urban environment tracked MBACs to a stronger extent than on LBACs (Supp Fig. 5). MBACs were additionally related to familial instances of Alzheimer’s and related dementias; which were not as relevant for LBACs. Social home environment, such as living with relatives, was more relevant for *how* brain asymmetry progresses (LBACs) than *how much* (MBACs). Importantly, performance on specific cognitive tasks for assessing fluid intelligence related to both LBACs and MBACs. Mental health phenotypes, and in particular mental health diagnoses, were reflective of LBACs and MBACs. Age and sex did not reflect longitudinal progression of brain asymmetry as strongly as these specific domains of lifestyle and behavior.

We also observed links between brain asymmetry changes to socioeconomic status indicators, cognitive functioning, and health. Socioeconomic status indicators, such as change in household income and home heating type, were linked to asymmetry pattern change (Fig. 3B). For example, directional change in asymmetry pattern 6 (LBAC) was the strongest regressor in evaluating change in total household income. Varied performance on fluid-intelligence questions across imaging visits was linked to brain asymmetry changes; in particular, assessments which involved language proficiency, such as word or concept interpolation. Finally, altered health landscapes of family members reveal links to changes in brain asymmetry. Overall, our findings point to a complex interplay between multifaceted aspects of life and the re-configuration of brain asymmetry patterns.

### Brain asymmetry changes explain diagnoses from health records

Motivated by cues from self-report health measures (cf. last section), we next capitalized on diagnoses from the linked electronic health record data of UK Biobank participants. To this end, we condensed a total of 4,448 ICD codes (both ICD9 and ICD 10) in the UK Biobank imaging cohort into 174 composite disease groups (cf. Methods) – naturally linked diagnosis groups. We then conducted a medical diagnosis-wide association study (MeDiWAS) linking brain asymmetry changes to clusters of physician-certified disease spanning 17 broad disease categories, ranging from congenital diseases, to respiratory or digestive illnesses, to malignant neoplasms.

We identified physician-diagnosed mental health disorders to be associated with within-individual changes in brain asymmetry across both methods of quantifying brain asymmetry changes (LBACs and MBACs). In particular, asymmetry pattern 12 LBACs and pattern 13 MBACs were associated with a disease cluster encapsulating depression and related diagnoses (major depressive disorder, suicide attempts and suicidal ideation; Fig. 3). These two brain asymmetry measures were weakly correlated to each other (Pearson’s r = −0.011, Supp Fig. 1C), and highlighted asymmetries in separate areas of the brain. Pattern 12 highlighted concurrent asymmetry shifts in ipsilateral lingual and anterior cingulate gyrus and contralateral superior frontal gyrus. Pattern 13 emphasized concurrent asymmetry shifts in postcentral gyrus, posterior middle temporal gyrus, and anterior supramarginal gyrus alongside contralateral shifts in asymmetry of angular gyrus and parietal operculum cortex. Pattern 12 LBACs, but not pattern 13 MBACs, were associated with a disease cluster implicating physician-diagnosed anxiety disorder diagnoses. Our previously noted relationship between self-reported mental health indicators and brain asymmetry changes (cf. previous section) was reflected by here-discovered associations between specific longitudinal whole-brain asymmetry changes and physician-diagnosed clinical mental health and psychological disorders.

**Figure 4:**
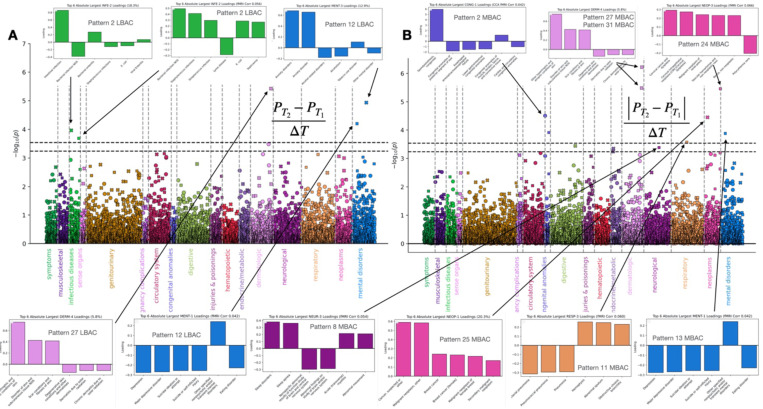
Diagnoses from physicians relate to measures of brain asymmetry change Manhattan plots relate (A) LBACs and (B) MBACs across the population to 174 composite medical diagnosis phenotypes spread across 17 domains. For each composite diagnosis phenotype, Pearson’s correlation coefficients are shown in units on logarithmic scale of the associated P value. Results from all 33 distinct asymmetry patterns are showcased. Horizontal lines indicate the significance thresholds at FDR labeled FDR, Bonferroni correction (0.05/174) labeled BON, with 5 and 6(LBAC) or 8 and 12 (MBAC) phenotypes passing each respective threshold. Composite medical diagnoses were constructed either by (i) PCA on 1,662 medical diagnosis phecodes from the full UKBB cohort (~500,000 individuals) (crosses) (ii) CCA on 1,447 medical diagnosis phecodes from the brain imaging UKBB cohort (~40,000 individuals) and resting state fMRI (circles) or (iii) PLS-C on 1,447 medical diagnosis phecodes from the brain imaging UKBB cohort (~40,000 individuals) and resting state fMRI (squares). Inlaid graphs showcase the 6 largest absolute medical diagnosis phecodes which contribute to the composite diagnosis phenotype. Text within inlaid graphs indicate which asymmetry pattern was significantly correlated to the composite diagnosis phenotype. Brain asymmetry changes are consistently related to common physician diagnosed mental health disorders including depression, suicidality and anxiety, substance use and sleep disorders.

Extending beyond the mental health disease category, the magnitude of changes in pattern 8, which highlights asymmetries in the temporal gyrus with particular emphasis on inferior temporal gyrus and temporal fusiform cortex, was linked to a disease cluster emphasizing sleep disorders, including insomnia (F51.X, G47.X, 307.4), and sleep apnea (G47.3) (Fig. 4). A separate category of diseases, dermatological disorders, emerged as significantly associated with measures of brain asymmetry change. Clusters within the dermatological disorder category were linked to both MBACs and LBACs in circumscribed brain asymmetry patterns. In particular, a cluster of dermatological conditions including ichthyosis, scleroderma, actinic keratosis, and scarring conditions including keloids was significantly associated to changes in asymmetry patterns 27 (LBAC and MBAC) and 31 (MBAC only) (Fig. 4). Asymmetry pattern 27 implicated the temporooccipital part of inferior temporal gyrus alongside ipsilateral asymmetry of posterior cingulate gyrus and contralateral asymmetry in superior parietal lobule. Asymmetry pattern 31 meanwhile implicated asymmetry in white matter tracts involved in linking areas of the limbic system, including uncinate fasciculus and cingulum cingulate gyrus. Skin scarring, which we here linked to circumscribed changes in whole-brain asymmetry patterns, is known to result in poorer emotional well-being and quality of life (Brown et al., 2008), while access to dermatological care more broadly has been linked to socioeconomic strata (Tripathi et al., 2018).

Certain disease categories were tightly linked to changes in particular asymmetry patterns. Asymmetry pattern 2, which links asymmetries in brain features including planum temporale, cerebral peduncle, corticospinal tract, and specific cerebellar lobules including lobules VI, VIIb, and VIIIa, was specifically associated with disease clusters containing infectious diseases. In particular, LBAC in asymmetry pattern 2 was linked with intestinal and bacterial infections, including staphococcus, streptococcus and septicemia (Fig. 4). In the analysis relating longitudinal changes in brain asymmetry to the aspects of behaviour which may track them (cf. Fig. 2A), LBACs in asymmetry pattern 2 was related to multiple measures of socioeconomic status, including homeownership status, educational qualifications.

Overall, we found that changes in the relative hemispheric balance of circumscribed sets of brain regions show coherent associations with physician-assigned medical diagnoses. These associations ranged from highly specific relations between particular asymmetry patterns and particular disease categories, to more broad associations between brain asymmetry changes in general and physician-diagnosed disease clusters, flagging the domains of mental health disorders, sleep disorders, and dermatological conditions.

## Discussion

The acquisition of new skills (e.g., learning a new language (Stein et al., 2012), how to juggle (Boyke et al., 2008; Draganski et al., 2004) or the development of existing ones (e.g., learning new words in a language you already know (Hofstetter et al., 2017), strengthening empathy skills (Valk et al., 2017)), have been shown to induce structural changes in the adult brain, even across short time spans of hours or weeks in boutique datasets. To expand this purview, we here examined brain-global changes in the brain hemispheres at a large breadth (population scale), depth (extensive phenotyping) and width (over the course of years). Across our analyses, the most important finding is that brain asymmetry is not static over time, taking into account brain-wide neuroanatomical features of the cerebellum, cortex, subcortex, and major white matter tracts. Rather, our findings attest to specific longitudinal *changes* in principled patterns of structural brain asymmetry that readily track hundreds of markers across lifestyle domains. Complementing this finding, we further revealed that structural brain asymmetry changes can reflect specified physician-diagnosed illnesses (ICD9/10 codes), including mental health disorders, like depression and anxiety, but also sleep disorders. Collectively, our findings establish an intimate link between life events and brain remodeling of hemispheric asymmetry.

To our knowledge, a focused study on the longitudinal progression of whole-brain asymmetry has not yet been done. In our view, one or several of the following recurring characteristics marked brain asymmetry research to date: (i) small sample size, (ii) cross-sectional study design, (iii) limited repertoire of available phenotype measurements, (iv) focus of analysis and interpretation on one anatomical region at a time, separately from the respective other brain parts, or v) consideration of a single kind of brain measurement modality. Until recently, neuroscientific endeavours have been conducted on cohorts of dozens of people on average (Szucs & Ioannidis, 2020; Woo et al., 2017). Within the field of hemispheric asymmetry, there exist a handful of studies examining cohorts over 1000 individuals (Kong et al., 2018; Kong et al., 2021; Kong et al., 2022; Saltoun et al., 2023; Zhao et al., 2022). Of these existing large sample size studies, all are limited to cross-sectional cohorts. Yet, longitudinal changes have been demonstrated to reveal stronger effects than cross-sectional analyses (Di Biase et al., 2023). Further, longitudinal research in general often uncovers biologically relevant rates of change in one hemisphere (cf. introduction). Here, we conducted a large-scale (n > 1,000) *longitudinal* study examining structural brain asymmetry, revealing progression of relative hemispheric structural balances which pervade all examined levels of brain organization. Comprehensive reviews have noted a tendency to examine one phenotype of interest in isolation of others (Vingerhoets, 2019); here, we systematically assessed hundreds of tangible behaviours and lifestyle factors, and changes therein. Moreover, examinations of structural asymmetry have often focused on either the cortical shell or subcortical structures, while neglecting cerebellar asymmetry (Kong et al., 2018; Kong et al., 2021; Kong et al., 2022; Zhao et al., 2022). Here, we concurrently considered brain asymmetry across cerebellar, cortical, subcortical, and white matter structures.

By overcoming such important shortcomings, our present study revealed complex, multifaceted dynamics of whole-brain structural brain asymmetry *change* manifesting over the course of years. Asymmetry pattern changes co-occurred across the population in ways that defy simple region-based explanations. Patterns emphasizing similar regions showcased diverging population-level rates of change, and patterns with similar population-level dynamics emphasize disparate regional asymmetries. These finding potentially point to underlying factors which have varied interrelationship with the evolving brain asymmetry.

Our results suggest that asymmetry change itself is biologically relevant. Despite a paucity of longitudinal research explicitly targeting hemispheric brain asymmetry change, existing longitudinal studies which focus on regional changes across the brain repeatedly link hemispherically unbalanced changes in brain structure to behaviours spanning social awareness (Valk et al., 2017), visuospatial awareness (Boyke et al., 2008; Draganski et al., 2004), and language (Stein et al., 2012). Here, we found structural asymmetry change to largely tie into markers of mental health, cognitive abilities, and environmental factors. One’s relationship with one’s inner social circle is amongst the most important social interaction for one’s psychological and physical well-being (Dunbar, 2018; Schurz et al., 2021). Time since last contact is strongly tied to emotional closeness, particularly for kin relationships (Roberts & Dunbar, 2011). Individuals within a shared household may thus represent ones strongest emotional connections as well as most frequently contacted individuals. Our results repeatedly highlight a relationship between longitudinal progression of brain asymmetry and household composition across patterns. Household composition and size were consistently more strongly related to *how* brain asymmetry changes (LBAC) than to *how much* asymmetry changes (MBAC). The frequency of family and friends visit was more strongly linked to amount of brain asymmetry changes (MBAC) rather than directional change (LBAC). Social interactions, and lack thereof, have previously been linked to Alzheimer’s disease and related dementia (Flicker, 2010; Fratiglioni et al., 2004; Shafighi et al., 2023). The link charted herein between social markers and brain asymmetry may thus illuminate one putative factor in the relationship between brain asymmetry and Alzheimer’s disease and other dementias previously observed (Cherbuin et al., 2010; Haxby et al., 1990; Kumfor et al., 2016; Wachinger et al., 2016). As a separate recurring theme, we found that demographic factors, such as age and sex, are more strongly linked to amount of brain asymmetry change, not directional change. By contrast, tangible behaviour and lifestyle markers, such as retirement and social interaction proxies, were more strongly linked to directional rather than magnitude of asymmetry changes.

Different shades of asymmetry patterns do not all tell the same story. Rather, particular brain patterns of distributed local changes provide unique insight into brain-behavioural coupling in the context of longitudinal progression of brain structure. For example, directional change in asymmetry pattern 2 was specifically tied to hospitalization for infectious disease in the intestine. A growing body of experimental animal studies link the gastrointestinal tract and its microbiome to the brain and its functioning (for reviews see (Cryan & Dinan, 2012; Cryan et al., 2019; Foster & McVey Neufeld, 2013; Nicholson et al., 2012). In mice, specific alterations to the intestinal microbiota, either through administration of antimicrobials or microbiotic transplantation, lead to changes in behaviour and, notably, to brain-derived neurotrophic factor, a key neuronal growth factor, expression in the brain. These changes were independent of the autonomic nervous system, gastrointestinal-specific neurotransmitters, or inflammation (Bercik et al., 2011). Though a growing topic of interest, research into the gut-brain axis in humans is relatively sparse in comparison to within animal models (Cryan et al., 2019). Directional change in pattern 19 was specifically tied to the act of retiring. Future work is needed to disentangle how distinct aspects of major life transitions, which have repercussions on social, environmental, psychological, physical aspects of life, relate to retirement-linked brain asymmetry changes, with perhaps special emphasis on brain regions implicated in asymmetry pattern 19, such as tapetum, temporal occipital fusiform gyrus, and anterior parahippocampal gyrus. Separate lines of inquiry into other major life events, including but not limited to parenthood, finishing university studies, moving to a new city, children leaving home (“empty nest syndrome”), and concurrent brain asymmetry changes can serve as a fruitful contrast to retirement-level results presented herein. Such research may provide insight into how experiencing another period of rapid (external) change reflects in observed (internal) brain changes.

In conclusion, evolutionarily uniquely evolved brain asymmetries appear to be strongly shaped by many non-genetic mechanisms (Badzakova-Trajkov et al., 2010; Carrion-Castillo et al., 2020; Corballis, 2009); neither are they static. By in-depth explorations into within-subject *changes* in asymmetries in brain morphology over the span of years, we revealed that structural asymmetry is a dynamic rather than inert property of the brain. Our results further revealed the intricate interdependences of external experiences with internal brain adaptations. We found that changes in hemispheric balance between brain regions are closely tied into health and disease: behaviour, cognitive performance, socioeconomic status, and lifestyle as well as mental and physical medical conditions. These connections in some cases exceeded the effect sizes obtained for pervasively studied demographic factors, such as age and sex. In light of the here disclosed multifaceted dynamics, our study emphasizes the relevance of asymmetry changes in understanding human behavior. Our study also highlights the necessity of longitudinal research in comprehending the brain’s adaptation to diverse life events.

## Methods

### Population Data Source

The UKBB is an epidemiological cohort initiative that offers extensive behavioral and demographic assessments, medical and cognitive measures, as well as biological samples for ~500,000 participants recruited from across Great Britain (https://www.ukbiobank.ac.uk/). This openly accessible population dataset aims to provide multimodal brain-imaging for ~100,000 individuals, in the years to come. Initial MRI brain scanning for participants began being collected in 2014 onwards. Repeat brain-imaging visits commenced in 2019. All participants provided informed consent. The present analyses were conducted under UK Biobank application number 25163. Further information on the consent procedure can be found here (biobank.ctsu.ox.ac.uk/crystal/field.cgi?id=200). Our present study was based on the data release from February 2020 that augmented brain scanning information to ~40,000 participants (48% male). Participants were aged 40–69 years when recruited (mean age 55, standard deviation [SD] 7.5 years). Of these ~40,000 participants, a subset of 1,425 (49% male) participants were scanned on two occasions that were 2–3 years apart.

In an attempt to improve comparability and reproducibility, our study built on the uniform data preprocessing pipelines designed and carried out by FMRIB, Oxford University, UK (Alfaro-Almagro et al., 2018). Participants in the UKBB underwent homogeneous data collection and processing pipelines as described before (Miller et al., 2016). Resultant brain imaging measures were made available to researchers in the form of expert-curated image-derived phenotypes (IDPs). Such IDPs span several complementary brain-imaging modalities. As our study aimed to specifically investigate structural asymmetries, we drew on carefully curated IDPs related to grey matter morphology captured by T1-weighted structural magnetic resonance imaging (sMRI) and white matter morphology captured by diffusion-weighted magnetic resonance imaging (dMRI). dMRI-derived measures, made available by the UKBB Imaging team, quantify microstructural aspects of major white matter connections (see Miller et al. (2016) for a review of dMRI modalities). Relying on common practices, we elected to use only one kind of dMRI-derived IDPs: fractional anisotropy (FA), which is typically described as a sensitive measure of white matter integrity (Miller et al., 2016). The final collection totalled to 184 anatomical target structures combined three different reference atlases with brain-derived information: i) 111 cortical and subcortical volumes (Harvard-Oxford atlas), ii) 48 major white matter tract integrities (John-Hopkins University atlas), and iii) 25 cerebellum lobe volumes (Diedrichsen atlas).

Data integrity and quality control was undertaken by the UKBB team. In our present study, participants who underwent two imaging scans were selected. Of these, any participant with no IDPs for a given modality of interest for either visit was discarded from downstream analysis. For example, any participant with sMRI IDPs related to region volume but no dMRI IDPs related to white matter FA was excluded from further analysis. In total, our dataset consisted of 1,425 participants (695 male) with biologically interpretable brain measures drawn from three commonly used anatomical reference atlases.

### Complementary Notions of Asymmetry Change

Quantification of individual-level structural brain asymmetry capitalized on previously-established whole-brain asymmetry patterns, which directly integrate structural asymmetries across cerebellum, cortex, subcortex and white matter structure into holistic measures of whole-brain structural asymmetries. Full details on the creation and are available in the work by Saltoun and colleagues (2023).

In brief, structural brain information are unified into 85 unitless lateralization indices capturing the relative left-right imbalance between homologous brain features. Mathematically, the lateralization index (LI) of each pair of corresponding atlas parcels was computed as the normalized difference between the right (R) and left (L) homologues according to the following formula: LI = (R-L) / (0.5*(R+L)). Once computed, the lateralization indices were normalized and corrected for technical deconfounding sources in accordance with Saltoun and colleagues (2023). Technical confound variables comprised head and body size, head motion at task, head motion at rest, head position in scanner, and scan site, following previous work on the UKBB (Schurz et al., 2021; Spreng et al., 2020). In the present study, normalization and technical deconfounding parameters were identical to those used in Saltoun and colleagues (2023), which were obtained from the baseline MRI scans of participant set of 37,441 individuals (17,537 male).

Once obtained, the nuisance-adjusted, z-score-transformed LI metrics were transformed into individual-level whole-brain asymmetry pattern expressions, according to the previously defined asymmetry pattern definitions (Saltoun et al., 2023). Mathematically, asymmetry patterns $P\in R^{n\times 33}$ are defined as P=A·V, where $A\in R^{n\times 85}$ is the LI metrics for all n = 1425 individuals, and $V\in R^{85\times 33}$ is the pre-defined, publicly available asymmetry pattern definitions. Asymmetry patterns V were originally obtained from singular value decomposition of the laterization indices for 37,441 individuals (Saltoun et al., 2023). In that previous work, only asymmetry patterns robust to noise were retained in the analyses. The top 33 most-explanatory asymmetry patterns were retained as these asymmetry patterns are robust to noise (Saltoun et al., 2023).

Thus, pattern expression P was here obtained on the basis of publicly available pattern definitions V and individual-level LI expression of 85 brain features. The procedure of obtaining asymmetry pattern expression P was carried out as described above identically for both time points, for a total of 33 asymmetry patterns for 1425 individuals at 2 timepoints per individual.

Within all individuals, the expression levels of a particular asymmetry pattern was compared across timepoints. That is, the expression strength of a given asymmetry pattern at baseline, $P_{1}$, was compared to the expression strength of the same asymmetry pattern in the same person at the follow-up visit $P_{2}$. To account for variations in the time intervening between subsequent scans, changes in asymmetry pattern were further adjusted for the number of days between visits to acquire a yearly rate of asymmetry pattern change. Overall, the longitudinal change in expression of an asymmetry pattern k in a person is given by the following expression

$$\text{Δ}P_{k}=\frac{P_{k_{2}}-P_{k_{1}}}{t_{\left[days\right]}}*365\;days/year$$

More precisely, the quantity $\text{Δ}P_{k}$ represents the lateralized brain asymmetry change (LBAC) of asymmetry pattern k, which captures the directional shift in brain asymmetry. That is, the LBAC quantifies instances where hemispheric homologs display larger left-hemispheric bias with time separately from instances where a larger right-hemispheric bias with time. As a complementary viewpoint, the degree of asymmetry was also captured in a measure that we referred to as magnitude of brain asymmetry change (MBAC). The MBAC quantifies instances where the brain becomes more structurally asymmetric (positive MBAC) separately from those where the brain becomes less structural asymmetric (negative MBAC). This is done without regard for whether, as an example, the increase of structural asymmetry was the result of existing left-biased structural asymmetries becoming more pronounced (increasing left-bias LBAC), or from a homologous pair that is symmetric at baseline later displaying an asymmetry corresponding to a larger right-hemisphere homolog (increasing right-bias LBAC). In the previous examples, two opposing directions of asymmetry change (LBAC) resulted in the same macroscopic trend of increased magnitude of brain asymmetry (MBAC).

Mathematically, these quantities are related to the quantity $\text{Δ}P_{k}$ described above as follows:

$${\text{LBAC}}_{\text{k}}=\text{Δ}P_{k}=\frac{P_{k_{2}}-P_{k_{1}}}{\text{Δ}t_{\left[days\right]}}*365\;days/year$$

and

$${\text{MBAC}}_{\text{k}}=\left|\text{Δ}P_{k}\right|=\frac{\left|P_{k_{2}}-P_{k_{1}}\right|}{\text{Δ}t_{\left[days\right]}}*365\;days/year$$

The two measures of brain asymmetry offer complementary viewpoints into the morphological asymmetry changes of particular brain features. The MBAC measure considers whether the total amount of asymmetry in the brain has increased or decreased, whereas the LBAC measure considers which hemisphere experienced larger relative shifts in brain volume (grey matter) or structural integrity (white matter). Across the population, these measures diverge from each other if, for example, asymmetry patterns may consistently become more pronounced over time (positive MBAC values indicating larger degrees of absolute asymmetry over time) but individually this asymmetry manifesting as a combination of some individuals whose asymmetry becomes more right-hemisphere biased while in others the brain becomes more left-hemisphere biased. Hence the two perspectives on brain asymmetry thus allowed us to disentangle *how* the brain sides change (LBAC) from *how much* the brain sides change (MBAC).

As a final point, the interpretability of LBAC measures was assisted by means of a reference measure. Broadly, LBACs were benchmarked such that positive-valued LBACs correspond to larger cortical grey matter decrease in the left hemisphere as compared to the right hemisphere. To this end, the cortical grey matter in each hemisphere was assessed by taking the sum of all 48 Harvard-Oxford atlas-identified cortical grey volumes for each hemisphere (96 total volumes) for each timepoint. The total hemispheric cortical grey matter volume ($G_{R}$ and $G_{L}$ for the right and left hemisphere respectively) at each time point was compared. The change in grey matter volume for each hemisphere was calculated as

$$\text{Δ}G_{H}=G_{H_{2}}-G_{H_{1}},$$

where $G_{H}$ is the cortical grey matter volume for a given hemisphere H, and $G_{H_{2}}$ is the cortical grey matter volume at timepoint 2 for hemisphere H. Once the change in cortical grey matter volume was obtained for both hemispheres, the relative rate of cortical grey matter volume change was assessed as following

$${\text{ΔG}}_{\text{Δ}}=\frac{\text{Δ}G_{R}-\text{Δ}G_{L}}{\text{Δ}t_{\left[days\right]}}*365\;days\text{/}year\text{,}$$

where ${\text{ΔG}}_{\text{Δ}}$ is a measure of the asymmetry of cortical grey matter volume change between hemispheres. If both hemispheres show enlargement of cortical grey matter volume over time(${\text{ΔG}}_{\text{R}}>0$ and ${\text{ΔG}}_{\text{L}}>0$) then the quantity ${\text{ΔG}}_{\text{Δ}}$ would be positive if the right hemisphere experience greater grey matter volume gains than the left hemisphere over the same period of time. If the cortical grey matter volume shrinks in both hemispheres over time (${\text{ΔG}}_{\text{R}}<0$ and ${\text{ΔG}}_{\text{L}}<0$), then the quantity ${\text{ΔG}}_{\text{Δ}}$ would be positive if the volume decline observed in the left cortex is faster than the volume decline observed in the right cortex (mathematically, if ${\text{ΔG}}_{\text{L}}$ is more negative than ${\text{ΔG}}_{\text{R}}$). This whole-cortex measure of relative hemispheric volume change serves as the yardstick we used to contextualize LBAC measures. The sign of LBAC measures was adjusted such that LBAC measures are positively correlated with a positive ${\text{ΔG}}_{\text{Δ}}$ quantity. That is, positive LBAC measures indicated faster cortical volume growth in the right hemisphere, or equivalently faster cortical volume decline in the left hemisphere.

### A Curated Portfolio of Target Behavioural Phenotypes

In order to ground our computed indices of LBACs and MBACs in potential real-world implications, we performed a rich annotation of the derived asymmetry pattern changes benefitting from a wide variety of almost 1,400 lifestyle factors, behavioural changes, demographic indicators, and mental health assessments. These ~1,400 indicators are subdivided into ~1,000 baseline behavioural phenotypes as well as ~400 behavioural changes which contrast behaviour and lifestyle at imaging visits 1 and 2.

Baseline behavioural phenotypes were extracted in the identical manner as described in previous work by Saltoun and colleagues (2023). As a broad overview, feature extraction for ‘baseline’ behaviour was carried out using two utilities designed to obtain, clean and normalize UKBB phenotype data according to predefined rules. Initially, a raw collection of ~15,000 phenotypes was fed into the FMRIB UKBB Normalisation, Parsing And Cleaning Kit (FUNPACK version 2.5.0; https://zenodo.org/record/4762700#.YQrpui2caJ8). FUNPACK was used to carefully curate a collection of phenotypes associated with the categories of interest and conduct data harmonization. The output of FUNPACK, consisting of ~3,300 phenotypes, was then input into PHEnome Scan Analysis Tool (PHESANT; (Millard et al., 2018), https://github.com/MRCIEU/PHESANT) for further refinement, cleaning and data categorization.The final set of 977 baseline phenotypes obtained from PHESANT was tested for systematic relations to the discovered asymmetry pattern changes (i.e., LBAC, MBAC) to explore possible relations between brain asymmetry change and behaviour.

More specifically, we used FUNPACK on the UKBB sample to extract phenotype information covering 11 major categories, ranging from lifestyle, to mental health and cognitive phenotypes to blood pressure measurements. These categories of interest were predefined in the FUNPACK utility through the -cfg fmrib arguments. They included only lifestyle phenotypes and excluded any brain-imaging-derived information. As there were only four phenotypes associated with diet, we discarded this category from downstream analysis. The FUNPACK setting which defined categories also contained built-in rules tailored to the UKBB. This tool performed automated refinements of the phenotype data, such as ruling out ‘do not know’ responses and replacing unasked dependent data. For example, a participant who indicated that they do not use mobile phones was not asked how long per week they spent using a mobile phone. In this case, FUNPACK would fill in a value of zero hours per week as a response to the latter question, though a value was not obtained at the time of assessment. FUNPACK’s thus built-in rules pipeline yielded 3,330 curated phenotype columns.

The FUNPACK output was then feed into PHESANT, a tool designed specifically for curating UKBB phenotypes (Millard et al., 2018), https://github.com/MRCIEU/PHESANT). The PHESANT toolkit combined phenotypes across visits, normalized, cleaned and categorized the data as belonging to one of four datatypes: categorical ordered, categorical unordered, binary and numerical. All categorical unordered columns were converted into binary columns to encode a single response. For example, the employment status phenotype was originally encoded as a set of values representing different conditions (e.g., retired, employed, on disability). Each of these conditions was converted into a binary column (e.g., retired true or false). The output of categorical one-hot encoding on unordered phenotypes was then combined with all measures classified by PHESANT as binary, numerical, or categorical ordered. The final set comprised carefully curated 977 phenotypes.

We deployed both FUNPACK and PHESANT with their default parameter choices regarding missing data on the full set of ~40,000 individuals with brain-imaging scans at the initial imaging visit. As a consequence, all columns with fewer than 500 participants were automatically discarded from further analysis as per PHESANT’s workflow protocols. Additionally, FUNPACK by default assessed pairwise correlation between phenotypes and discarded all but one phenotype of a set of highly correlated (>0.99 Pearson’s correlation rho) phenotypes. For example, left and right leg fat percentages were highly correlated (Pearson’s rho 0.992). Hence, only right leg fat percentage was included in the final set of phenotypes. The choices which phenotypes to discard were also automatically streamlined and conducted by FUNPACK.

### Change of Target Behavioural Phenotypes

To complement our measures of longitudinal *change* in brain morphology (cf. above), we aimed to also create measures of intra-subject *change* in our behavioral phenotypes (henceforth, behaviour *change*). Feature extraction for behavioural ‘change’ phenotypes underwent a similar procedure as for ‘baseline’ behavioral indicators. Measures of behavioural *change* were obtained for behaviours and phenotypes which measured at both imaging visits in the same subject. As before, both FUNPACK and PHESANT were utilized in conjunction, with the same set of categories extracted as for ‘baseline’ behaviour. However, slight adjustments were necessary into order to enable the comparison of behavioural response variables across time. Therefore, a number of steps, detailed below, were taken to ensure that behavioural responses from imaging visit 1 are comparable to behavioural responses from imaging visit 2 in the UK Biobank imaging cohort.

Part of PHESANT’s standard pipeline is the integration of behavioural responses across timepoints. As our goal was to contrast a snapshot of behaviour at imaging visit 1 with a snapshot of behaviour at imaging visit 2, we separately fed imaging visit 1 behaviour and visit 2 behaviour into PHESANT. FUNPACK was used to extract phenotypes collected at each respective visit prior to being fed into PHESANT. A separate feature of PHESANT’s standard pipeline is the automatic normalization applied to categorical variables. As our goal was to compare phenotypes at visit 1 and 2, normalization procedures applied separately to each visit may obfuscate behavioural change. Hence, PHESANT’s normalization procedure was suppressed using the ‘standardise=FALSE’ argument, with manual checks to verify that input values matched output variables after PHESANT was run. A third consideration in ensuring comparability between behavioural phenotypes at each visit related to PHESANT’s automatic categorization of phenotypes into categorical (ordered), categorical (unordered), binary, or continuous. For some phenotypes, the behaviour at one time point was categorized as categorical (ordered or unordered) but binary in another timepoint. In instances where different encoding was used on the same behaviour from different timepoints, behaviour from timepoint 2 was manually recoded according to rules PHESANT automatically applied to behaviour at timepoint 1.

Once behaviour at each timepoint was acquired, a measure of behavioural change was acquired through the following:

$$\text{ΔΦ}={\text{Φ}}_{2}-{\text{Φ}}_{1},$$

where ΔΦ is the behaviour change of phenotype Φ, and ${\text{Φ}}_{2}$ and ${\text{Φ}}_{1}$ are the behaviour as recorded at imaging visit 2 and 1 respectively. In the case of binary or categorical phenotypes, a value of ‘0’ indicates no change in behaviour between the two imaging visits. A value of ‘1’ indicates a behaviour which was present at timepoint 2 but not at timepoint 1 (for example, a non-smoker at timepoint 1 who smokes at timepoint 2). A value of ‘−1’ indicates a behaviour which was present at timepoint 1 but absent at timepoint 2. Normalization to mean 0 and standard deviation 1 was conducted on behavioural change phenotypes for all PHESANT-tagged continuous behaviours. As the total number of participants in the present study was 1,425 individuals, PHESANT’s native workflow step to remove all behaviours with fewer than 500 participants was suppressed. Behaviours with fewer than 10% response rate at either timepoint were dropped. In so doing, a total of 397 behavioural change phenotypes spanning 9 domains, were extracted through this procedure.

### Exploration of Asymmetry Change Linked to Life and Disease

To get a snapshot of how various aspects of life and behaviour track whole-brain asymmetry changes, we related a given domain of lifestyle or lifestyle change to a measure of brain asymmetry change. To this end, we regressed a given brain asymmetry change (LBAC or MBAC) onto phenotypes from a particular lifestyle domain. To disentangle the impact of common demographic factors from behavioural phenotypes, 6 demographic features (age, sex, age^2^, age*sex, sex*age^2^, time between visits measured in days) were included in all regressions.

$$\text{LBAC}={\text{β}}_{0}+\sum_{F\in \text{Demographics}}{\text{β}}_{\text{F}}*\text{F}+\sum_{D\in Domain}{\text{β}}_{{\text{ΔΦ}}_{\text{D}}}*{\text{ΔΦ}}_{\text{D}}$$

and

$$\text{MBAC}={\text{β}}_{0}+\sum_{F\in Demographics}{\text{β}}_{\text{F}}*\text{F}+\sum_{D\in Domain}{\text{β}}_{{\text{ΔΦ}}_{\text{D}}}*{\text{ΔΦ}}_{\text{D}},$$

where F is the full collection of 6 demographic features (age, sex, age^^^2, age*sex, age^^^2*sex, time between visits) and D is the set of phenotypes belonging to either a single baseline lifestyle domain, or a single lifestyle *change* domain. There are a total of 20 groups of analysis (11 baseline domains and 9 *change* domains).

For all regression analyses, the coefficient of determination, $R^{2}$, was used as an established performance metric to quantify the amount of variance in brain asymmetry change (model outcome variable) that can be explained by the lifestyle domain at hand (model input variables). As the number of behaviours within a domain depends on the domain at hand, we adjusted the resultant coefficient of determination to account for the number of features in the regression model as follows

$$R^{2}=R_{0}^{2}\frac{n-1}{n-d-1},$$

where $R_{0}^{2}$ is the unadjusted coefficient of determination, n is the number of participants, and d is the number of regression features. This formulation is called the *adjusted* R2 *statistic* and is widely used when comparing models of different size (James et al., 2013, p. Section 6.1.3).

The results from estimating these linear regression model specifications provide a broad snapshot of how broad lifestyle domains relate to brain asymmetry changes. They were conducted based on the 33 measures (i.e., number of previously reported brain asymmetry patterns) of directional brain asymmetry change (LBAC) and amount of brain asymmetry change (MBAC). A final set of regression anlayses without any lifestyle domain (in other words, with only 6 demographic features and intercept as regressors) was carried out – as a null baseline model without behavior effects to compare against.

We next sought to establish a complementary viewpoint into the relationship between brain asymmetry change and tangible behaviour changes. To this end, we conducted a series of regression analyses which related a given behavioural change to underlying brain asymmetry changes and demographic factors.


$$\text{ΔΦ}={\text{β}}_{0}+\sum_{F\in Demographics}{\text{β}}_{\text{F}}*\text{F}+\sum_{i=1}^{33}\;{\text{β}}_{{\text{LBAC}}_{\text{i}}}*{\text{LBAC}}_{\text{i}}+\sum_{i=1}^{33}\;{\text{β}}_{{\text{MBAC}}_{\text{i}}}*{\text{MBAC}}_{\text{i}}$$

As above, we included a set of 6 demographic variables, which include the time between visits, as covariates within the model. For maximum comparability between linear regressions obtained from this set of analysis to the above set, we reported adjusted R2 statistic as calculated above.

Behavioural changes have not been corrected for time between visits, and may be strongly related to either age or sex. Therefore we expected that the included demographic features (age, sex, time between visits) may have an outsized impact on the linear regression. To account for this, we aimed to determine the expected adjusted R2 statistic if there was no impact of brain asymmetry change on the behavioural change at hand (label shuffling permutation test). To this end, we have repeated the regression analyses after permuting LBAC and MBAC labels, which breaks the relationship between brain asymmetry change and the behavioural change at hand. The adjusted R2 statistic which resulted from these regressions was subtracted out from the adjusted R2 statistic from the unperturbed data. In so doing, we were able to quantify the added benefit of brain asymmetry changes (both LBACs and MBACs) on charting the relationship between behavioural change and brain and demographics.

For visualization purposes, the largest absolute beta coefficient from the above equation was extracted for each of the 397 separate behavioural change variables, each of which has one regression analysis linking brain change to behaviour change. The feature corresponding to this beta coefficient represents the strongest relationship between the behavioural change of interest and all examined input features. To ensure comparability of beta coefficients, all input features were normalized to mean 0 and standard deviation 1 prior to conducting the regression. The strongest feature was grouped according to membership of LBAC features, MBAC features, or demographic/intercept term features.

### Electronic Healthcare Record Data

To offer a distinct window into a separate field of biological phenotypes which may be linked to morphological between brain asymmetry pattern changes, we turned our attention to physician-diagnosed medical illnesses as captured through electronic health records. To do so, we performed a rich annotation of the derived brain asymmetry pattern changes by means of a phenome-wide association analysis benefitting from a wide variety of almost 1,700 disease phecodes extracted from participants’ electronic health records, spanning over 11,000 ICD codes. Feature extraction for both ICD-9 and ICD-10 diagnoses was carried out using the FMRIB UKBB Normalisation, Parsing And Cleaning Kit (FUNPACK version 2.5.0; https://zenodo.org/record/4762700#.YQrpui2caJ8). Once extracted, ICD-9 and ICD-10 codes were grouped into ~1,700 hierarchical phenotypes (phecodes) which combine related ICD-9 and ICD-10 codes into a single ‘phecode’, using previously established phecode definitions which span 17 disease classes (Wu et al., 2019). For technical reasons, the full set of ~1,700 disease phecodes were condensed using several data compression protocols on a per disease class basis, to create a total of 174 composite disease clusters. These composite disease clusters were finally compared to measures of brain asymmetry pattern change to probe for relations between morphological brain asymmetry changes and clinical diagnoses.

More concretely, we used FUNPACK on the full UKBB cohort (n = 502,507) to extract phenotype information related to ICD-9 diagnosis codes and ICD-10 diagnosis codes. This collection of 502,507 participants contained within it the full imaging cohort ( n ~40,000) and the repeat imaging cohort (n = 1,425). The FUNPACK utility contained built-in rules tailored to the UKBB which enabled the extraction of a total of 2,837 columns associated with ICD-9 codes (2,506 unique codes) and 14,217 columns associated with ICD-10 codes (8,845 unique codes).These ICD codes were collected on the basis of electronic health records linked to each participant. ICD-10 codes contain more granular information than ICD-9 codes (Wu et al., 2019); separate ICD-9 and −10 codes may reflect common etiologies (Denny et al., 2013; Denny et al., 2010). To consolidate related medical diagnoses across and within ICD coding paradigms, we used the Phecode Map 1.2 with ICD-10 codes made available from Wu and colleagues (https://phewascatalog.org/phecodes_icd10 (Wu et al., 2019)). In total, applying phecode definitions linking ICD codes resulted in a final set of 1,662 clinical phecodes obtained from 502,507 participants. These clinical phecodes spanned 17 disparate disease classes, ranging from congenital anomalies, to neoplasms, to mental disorders, to infectious diseases (Wu et al., 2019).

For technical reasons, the collection of 1,662 clinical phecodes required a further refinement prior to analysis with our set of whole-brain asymmetry changes. To this end, 3 separate data compression techniques were used to reduce the total set of 1,662 clinical phecodes into a set of 174 *composite disease clusters* which capture distinct underlying trends in the overall diagnosis landscape. For increased interpretability, these composite disease clusters each integrate disease status from phecodes within one disease class only.

As the first data reduction technique, we turned to principal component analysis (PCA), which is a commonly-used data reduction technique. PCA is an unsupervised method whose extracted latent factors represent the dimensions of maximum variance within the dataset. We applied PCA on the larger full UKBB cohort (n > 500,000), rather than the repeat imaging cohort (n = 1,425), to improve the representativeness and stability of the resultant composite disease clusters. To boost interpretability of resultant latent factors, PCA was applied separately to each of the 17 disease classes. In other words, PCA was applied 17 times, with each PCA application being specific to phecodes within a single disease class. The interpretability of latent factors was thus boosted because each latent factor is intrinsically linked to only one aspect of health. For example, the latent factor obtained from applying PCA to phecodes in the mental health disorders disease class represents clinical diagnoses within the mental health domain only, irrespective of diagnoses in other domains. This enables an at-a-glance look into the content captured by a given latent factor based on which disease category it captures. By contrast, PCA applied on the full set of 1,662 clinical phecodes at once would combine phecodes from across disease classes, which may make it more difficult to separate the effects of diseases in one class, such as mental health disorders, from diseases in another class, such as endocrine or metabolic disorders.

For each of the 17 disease clusters, the question of how many latent factors to keep was addressed by means of an empirical permutation analysis. To break the link between related disease phecodes, we perturbed each of the clinical phecodes within a disease cluster separately, before applying PCA on the permuted dataset. This enabled us to establish the expected explained variance in a scenario where there is no relationship between separate disease phecodes. A total of 5 permutations was done for each disease category, and the mean explained variance for each noise-based latent factor was taken as a reference measure. Unperturbed (true) latent factors were kept if their explained variance exceeded the explained variance of the equivalent noise-based latent factor. This procedure resulted in a total of 58 latent factors spanning 17 disease classes being retained, with the per-class latent factors ranging from one, in the case of the pregnancy complications and congenital anomality disease classes, to ten, in the case of genitourinary diseases (Table 1). On average, the retained latent factors explained 45.1% of the variance within their respective disease class (Table 1).

To complement the PCA-derived composite disease clusters, we used two doubly multivariate latent factor decomposition methods to establish latent factors which explicitly link disease status to brain activity. Specifically, we used canonical correlation analysis (CCA) and canonical partial least squares (PLS-C) decomposition. Both these self-supervised methods are doubly multivariate, meaning that they integrate information from one ‘block’ of variables, in this case disease status, with another ‘block’ of variables. We capitalize on the resting-state fMRI data available through the UKBB to establish composite disease clusters which link resting-state brain activity (one ‘block’) to clinical phecodes (the second ‘block’). Resting-state functional imaging was acquired over the course of 6 minutes for 37,526 participants. For technical reasons to avoid overfitting, we reduced the expert-curated full and partial correlation matrices (dimensions 25 and 100) made available by the UKBB team to 100 top PC components prior to applying dimensionality reduction techniques, in accordance with standard practice. As with PCA, both CCA and PLS-C were conducted on each disease class separately. The number of latent factors kept for each technique was equivalent to the number of latent components identified in the PCA analysis. That is, if the PCA analysis resulted in 3 latent factors for a given disease class, the top 3 CCA and top 3 PLS-C latent factors for that disease class were kept. In this way, we extracted an additional set of 116 composite disease clusters (58 each for CCA and PLS-C) from our original set of clinical phecodes. These composite disease clusters linked resting state brain activity (as captured in fMRI imaging) to disease status (as captured in clinical phecodes), and thus represented a different dimension of disease.

Finally, we charted robust cross-links between the subject-wise expression of a given brain asymmetry pattern change and the portfolio of 174 composite disease clusters, with appropriate correction for multiple comparisons. For each composite disease clusters, the Pearson’s correlation between the given composite disease clusters and the inter-individual variation in brain asymmetry change revealed both the association strength and accompanying statistical significance of the given disease cluster-brain asymmetry change association. For each uncovered brain asymmetry pattern change, two standard tests were used to adjust for the multitude of associations being assessed. First, we used Bonferroni’s correction for multiple comparisons, adjusting for the number of tested phenotypes (0.05/174 = 2.87e-4). Second, we further evaluated the significance of our correlation strength using the false discovery rate (FDR), another popular method of multiple comparison correction which is less stringent than Bonferroni’s correction. The false discovery rate (Benjamini & Hochberg, 1995) was set as 5% (Miller et al., 2016; Raizada et al., 2008; Sha et al., 2021) and computed for each pain archetype in accordance with standard practice (Genovese et al., 2002). For visualization purposes, disease clusters in Manhattan plots were coloured and grouped according to the category membership defined by the phecode map developed by Wu and colleagues (2019) and shape in accordance to the dimension reduction technique applied. This procedure was applied for measures capturing direction of brain asymmetry change (LBACs) as well as magnitude of brain asymmetry change (MBACs).

## Figures and Tables

**Table 1:** Disease class size before and after PCA-based data compression. Composite disease clusters (latent factors) are retained for latent factors whose explained variance exceed the explained variance of equivalent noise-based latent factors. PCA was applied separately for each disease class.

Disease Category	Number of Phecodes	Number of latent factors	Total Explained Variance of latent factors (%)
Circulatory System	155	4	50.1
Congenital Anomalies	54	1	38.3
Dermatological	94	4	45.3
Digestive	155	6	48.3
Endocrine/Metabo lic	146	2	51.5
Genitourinary	156	10	52.1
Hematopoietic	54	3	64.4
Infectious Diseases	55	2	44.7
Injuries and Poisonings	125	2	21.6
Mental Disorders	70	4	64.5
Musculoskeletal	120	2	36.0
Neoplasms	132	3	39.9
Neurological	78	5	53.5
Pregnancy Complications	44	1	22.9
Respiratory	75	6	64.0
Sense Organs	113	1	23.0
Symptoms	36	2	46.0
**Total**	**1,662**	**58**	**45.1 (Mean)**
